# Comparison between Analgesia Nociception Index (ANI) and self-reported measures for diagnosing pain in conscious individuals: a systematic review and meta-analysis

**DOI:** 10.1038/s41598-022-06993-z

**Published:** 2022-02-21

**Authors:** Daniela Abrão Baroni, Lucas Guimarães Abreu, Saul Martins Paiva, Luciane Rezende Costa

**Affiliations:** 1 Dentistry Graduate Program, Faculty of Dentistry, Universidade Federal de Goiás (UFG), Goiânia, Goiás Brazil; 2 Department of Paediatric Dentistry, Faculty of Dentistry, Universidade Federal de Minas Gerais (UFMG), Belo Horizonte, Minas Gerais Brazil; 3 Faculty of Dentistry, Universidade Federal de Goiás (UFG), Goiânia, Goiás Brazil

**Keywords:** Health care, Medical research, Signs and symptoms

## Abstract

The Analgesia Nociception Index (ANI), an objective measure of pain based on heart rate variability (HRV), has its usefulness in awake patients still unclear. This systematic review and meta-analysis aimed to assess ANI's accuracy compared to self-reported pain measures in conscious individuals undergoing medical procedures or painful stimuli. PubMed, Ovid, Web of Science, Scopus, Embase, and grey literature were searched until March 2021. Of the 832 identified citations, 16 studies complied with the eligibility criteria. A meta-analysis including nine studies demonstrated a weak negative correlation between ANI and NRS for pain assessment in individuals in the post-anesthetic recovery room (r = − 0.0984, 95% CI = − 0.397 to 0.220, I^2^ = 95.82%), or in those submitted to electrical stimulus (r = − 0.089; 95% CI = − 0.390 to 0.228, I^2^ = 0%). The evidence to use ANI in conscious individuals is weak compared to self-report measures of pain, yet ANI explains a part of self-report. Therefore, some individuals may be benefited from the use of ANI during procedures or in the immediate postoperative period.

## Introduction

The reliability of an instrument, test, or exam relies on its accuracy compared to the 'gold standard' for diagnosing a condition or disease. It is not different when pain is assessed. Self-reported measures, the gold standard in pain measurement, allow for evaluations in patients without neurological impairments, conscious and awake individuals, or those with sufficient cognitive development to report their perceptions of pain through scales, questionnaires or interviews^1–4^.

On the other hand, assessments of nociception combined with the best pain control strategy (analgesia) have encouraged studies with instruments that can evaluate pain objectively^5^. In this perspective, the Analgesia Nociception Index (ANI, Physiodoloris™; Metrodoloris, France) is a non-invasive tool placed on the market in the last decade. ANI is based on the analysis of the respiratory fluctuations of heart rate^6,7^.

The pain/analgesia evaluation algorithm^8^ was idealised and used in patients submitted to different procedures under general anaesthesia^9–13^ to assess autonomic nervous system (ANS) activity and thus optimise analgesic drugs prescription^14,15^. ANI analyses the balance of nociception/antinociception through heart rate variability (HRV) on a scale from 0 (maximum of nociception/predominance of the sympathetic nervous system) to 100 (complete analgesia/predominance of the parasympathetic nervous system), making a distinction between appropriate and inappropriate antinociception in anesthetised adult patients^16–21^.

Nevertheless, ANI has also been used in conscious patients because of its understandable mechanism, easy reading, and non-invasive characteristic^22,23^. Therefore, an overview of the results obtained by comparisons with self-reported pain tools could help define its accuracy.

The level of evidence to support the application of the ANI technology in awake patients is still unclear. Given that there is no standardisation in clinical references in the literature, this systematic review and meta-analysis aimed to compare ANI and self-reported measures for diagnosing pain in conscious individuals undergoing medical procedures or painful stimuli.

## Methods

### Protocol and registration

This systematic review and meta-analysis conform to the Preferred Reporting Items for Systematic Reviews and Meta-Analysis (PRISMA) checklist^24^. A protocol was drafted and registered in the International Prospective Register of Systematic Reviews (PROSPERO, CRD42018114439).

### Eligibility criteria

Observational studies in which ANI was compared with any subjective measures (numerical scales or questionnaires) to assess pain in awake or conscious individuals undergoing medical procedures or painful stimuli were included. No restrictions regarding the age of participants and the study's language or year of publication were imposed. Studies reporting assessments of individuals with cognitive or neurological impairment were excluded. So were letters to the editor, meeting abstracts, and qualitative studies.

The following PIRD^25^ acronym was applied:

*Population:* conscious individuals undergoing medical procedures or painful stimuli.

*Index Test:* ANI.

*Reference test:* self-reported measures of pain.

*Diagnosis of interest:* pain.

### Information sources

Computerised searches across five electronic databases were conducted in October 2018. The databases used were PubMed (National Library of Medicine), Scopus (Elsevier), Web of Science (Clarivate Analytics), Ovid (Wolters Kluwer), and Embase (Elsevier). An update took place in March 2021. In addition, the reference lists of the included articles were also screened for references that might not have been retrieved during the computerised searches. Finally, searches for literature in Open Grey and Google Scholar were undertaken; the searches were limited to the first 300 most relevant hits^26^. Duplicate references were removed upon identification. The references were managed using EndNote software (Thomson Reuters, Toronto, Canada; https://www.myendnoteweb.com).

### Search strategy

The search strategy in PubMed, Ovid, Embase, and Web of Science was analgesia nociception index OR analgesia-nociception index. A specific search strategy was tailored for Scopus: "analgesia nociception index" OR "analgesia-nociception index", and for Embase: 'analgesia nociception index'/exp OR 'analgesia nociception index'. Searches in Google Scholar and Open Grey were carried out with the "analgesia nociception index OR analgesia-nociception index" algorithm.

### Study selection

Study selection was conducted in two phases. In Phase 1, two review authors (DAB and LRC) read the titles/abstracts independently. The references whose titles/abstracts met the eligibility criteria were included straight away. In Phase 2, the same authors evaluated the full references with titles/abstracts containing insufficient information for a final decision. The references whose full texts met the eligibility criteria were also included. In both phases, divergences between authors were resolved by discussion until a consensus was reached.

### Data extraction and data items

Two review authors (DAB, LRC) performed data extraction independently. Disagreements were resolved through discussion. If disagreements persisted, a third review author (LGA) decided. When additional or missing information was needed, the authors of the articles were contacted. The primary data were extracted and are cited in Table 1.

**Table 1:** Summary of characteristics and results of the included studies.

Author(s), year, country, language	Participants, study design, and period of data collection	Pain subjective measure	Pain objective measure evaluation	Health procedures and anaesthetics/painful stimuli	Main results
Le Guen et al.^22^ (2012), France, English	Initial sample size: not reported, Parturients > 35 weeks of gestation, ASA-I before epidural analgesia, Prospective observational study, period of data collection not reported	Self-reported VAS every 5 min	ANI (PhysioDoloris monitor) recorded every 5 min (simultaneously)	Labour without regard to uterine contractions, No anaesthetics	Final sample size: 45 parturients Linear regression: r^2^ = − 0.179 ± 0.032(SEM), *p* < 0.0001, between ANI and VAS. Between contractions: regression coefficient = − 0.10 ± 0.04, *p* = 0.007, 40.7% of variability explained; During contractions: r = − 0.36 ± 0.10, *p* = 0.0006, 44.5% of variability explained VAS > 30 and ANI = 49: PPV 70% (95% CI 57–83) and NPV 78% (95% CI 66–90)
Boselli et al.^30^ (2013), France, English	Initial sample size: not reported, Patients ASA I–II 18–75 years, Prospective observational study June-July 2012	Self-reported NRS reported 10 min after arrival in PACU and at the end of PACU stay	ANI (PhysioDoloris monitor) recorded in the PACU on arrival in PACU and at the end of PACU stay	Endoscopy, otolaryngology, or plastic surgery, General anesthesia Halogenated or propofol	Final sample size: 200 patients Linear regression: negative linear relationship between ANI and NRS: ANI − 5.2 versus NRS + 77.9, r^2^ = 0.41, *p* < 0.05; NRS > 3 and ANI performance: AUC = 0.86, 95% CI (0.8–0.91) Propofol: AUC = 0.93, 95% CI (0.85–0.97) Halogenated: AUC = 0.82, 95% CI (0.73–0.88); ANI ≤ 57 = threshold for moderate pain – sensitivity and specificity (95% CI) to discriminate between NRS ≤ 3 and NRS > 3 were 78% (66–87) and 80% (73–87), respectively; PPV 67 (56–77); NPV 88 (80–93) ANI predicting severe pain (NRS ≥ 7): AUC = 0.91, 95% CI 0.86–0.95; sensitivity and specificity (95% CI) were 92% (62–100) and 82% (76–88) respectively; PPV 25 (13–41); NPV 99 (97–100)
Ledowisk et al.^16^ (2013), Australia, English	Initial sample size: 120 adults (mean age: 35 years), Prospective observational study, Period of data collection not reported	Self-reported NRS every 5 or 10 min in PACU	ANI (PhysioDoloris monitor) recorded every 5 or 10 min in PACU preceding NRS	Non-emergency surgery: plastic, orthopaedic, general and others, Sevoflurane and fentanyl	Final sample size: 114 patients Spearman Correlation: (r = − 0.075;P = 0.034); negative, small correlation between ANI and NRS ANI was higher in states of deep sedation compared with full consciousness [mean (SE): 73.4 (14.6) vs 58.7 (15.1); P < 0.001]; - comparing the extremes of pain (mean (SE): NRS 0 = 63 (1.4) vs. NRS 6–10 = 59 (1.4) P = 0.027; ANI scores before 52 (14) and 5 min after a bolus of fentanyl 54 (15) did not differ (p > 0.05); ANI scores did not differ between different categories of NRS, except for NRS 6–10 = 59.2 (1.5) when compared with NRS 0 = 62.9 (1.4), with AUC = 0.434. Sensitivity and specificity of ANI around 50%
Boselli et al.^31^ (2014), France, English	Initial sample size: 297 individuals ASA I–II 18–75 years, Prospective observational study, October 2012-April 2013	Self-reported NRS administered within 10 min of arrival in PACU	ANI (PhysioDoloris monitor) recorded immediately before tracheal extubation	Otolaryngology or orthopaedic surgery, General anesthesia: Induction: EV ketamine, propofol and remifentanil Maintenance: sevoflurane or desflurane In some cases: regional anaesthesia, cisatracurium as a muscle relaxant	Final sample size: 200 patients Linear regression: r^2^ = 0.33, *p* < 0.01; negative linear relationship between ANI and NRS ANI = 68.1–4.2 versus NRS, Mean (SD) ANI values were higher (*P* < 0.01) between patients with initial NRS ≤ 3 = 68 (18) and NRS > 3 = 42 (12), NRS > 3 and ANI performance: AUC = 0.89, 95% CI 0.84–0.93 Orthopedic surgery: AUC = 0.93, 95% CI 0.86–0.97; Otolaryngology: AUC = 0.83, 95% CI 0.75–0.90 ANI < 50 = threshold to predict pain – sensitivity and specificity (95% CI) to discriminate between NRS ≤ 3 and NRS > 3 were 86% (75–93) and 86% (79–92), with 77% (66–89) positive predictive value and 92% (85–96) negative predictive value
Jeanne et al.^42^ (2014), France, English	Initial sample size: 30 adults patients (median age 68), ASA I–II, Prospective observational study, Period of data collection not reported	Self-reported VAS after the end of surgery in PACU when the patient's claimed pain (VAS ≥ 50) and after the suppression of pain (VAS ˂ 10)	ANI (PhysioDoloris) ) recorded continuously	Orthopaedic surgery of total knee replacement, General anesthesia: Propofol and sufentanil Premedicated with midazolam (0.05 mg/kg) orally 1 h before the start of surgery, Propofol and sufentanil	Final sample size: 27 A ROC analysis showed poor predictability of pain in conscious patients, with an area under the surface of 0.65 and a "best fitting" threshold of 64 (sensitivity = 61%; specificity = 65%). No correlation was evidenced between ANI and VAS scores (Spearman rank test, r^2^ = − 0.164, P = 0.25)
Jess et al.^33^ (2016), Germany, English	Initial sample size: 20 healthy male students (mean age 24.2 years), Single-blinded, randomised crossover study, Period of data collection not reported	Self-reported in nin a single session after each stimulus: - electrical unexpected painful stimulus (UPS) - electrical expected painful stimulus (EPS) - neutral nonpainful stimulus (NPS) - placebo stimulus	ANI (ANI Monitor) recorded continuously	Baseline measure with no disturbance followed by four stimuli applied in random order on the right forearm (unexpected and expected electrical pain, expected nonpainful and sham stimuli) Each stimulus followed by a recovery time of 5 min; No analgesics, sedatives, or anaesthetics	Final sample size: 20 students ANI decreased after random stimulus (maximal decrease of 25.0%, SD 7.3) and did not allow differentiation of painful, nonpainful, or sham stimuli in alert volunteers; Spearman correlation: (r = − 0.09, P = 0.60) ANI minimum and NRS showed no correlation
Papaioannou et al.^34^ (2016), France, English	Initial sample size: 20 conscious adults 17–75 years, with partial or full-thickness burns, Prospective observational study, January–June 2014	Self-reported NRS evaluated before starting the procedure, and each time the patient-perceived pain	ANI (PhysioDoloris) recorded continuously, CARDEAN (Phillips MP50 monitor) recorded continuously	Wound treatment procedures, Morphine and ketamine before the procedure, plus morphine and sufentanil during the procedure at the discretion of the anesthesiologist	Final sample size: 20 adults ROC curve: AUC = 0.7559 SE (0.004); IC 0.747–0.764 Sensitivity = 67%,Specificity = 70%, PPV = 0.36, NPV = 0.89 Significant decrease in ANI values between time points with no pain (NRS: 0, 66.74 ± 21.99) and upon nociception (NRS: 1–10, 50.37 ± 16.90, p < 0.05), As well as between time points with different pain intensities (low pain with NRS: 1–3, 52.57 ± 15.13 vs. moderate/severe pain with NRS: 4–10, 46.83 ± 18.86, p < 0.05, respectively. Wilcoxon and Kruskal–Wallis tests
Xie et al.^41^ (2016), China, Chinese	Initial sample size: 80 conscious patients 21–77 years, ASA I–III Prospective observational study, Period of data collection not reported	Self-reported NRS evaluated after entering the PACU, patient with spontaneous breathing and consciousness (T0), after 10 min (T1); after 5 min (T2)	ANI (PhysioDoloris) was recorded at T0, T1, and T2	Elective surgery: Orthopedics, Gynecology, Stomatology and General Surgeries, General anaesthesia: Fentanyl and propofol/remifentanil Maintenance: inhalation of 1% to 2% sevoflurane	Final sample size: 74 patients Pearson correlation: r = − 0.705 (P < 0.05) AUC = 0.873, 95% CI (0.816–0.929) Sensitivity = 74.8%, Specificity = 87.5%, T0- AUC = 0. 817, 95% CI (0.727—0.907) T1—AUC = 0.819, 95% CI (0.733—0.906) T2—AUC = 0.940, 95% CI (0.902–0.979) ANI value is negatively correlated with NRS score
Issa et al.^41^ (2017), Canada, English	Initial sample size: 23 healthy volunteers 18–80 years, Prospective observational study, October- December 2014	Self-reported NRS every minute	ANI (PhysioDoloris) recorded continuously	Electrical stimulus at the wrist with increasing current intensity from 0 to 30 mA (5 mA increments, kept constant for three minutes at each level)	Final sample size: 23 volunteers Pearson correlation: (r = − 0.089; 95% CI − 0.19 to − 0.01; P = 0.045). NRS and ANI-mean: very weak negative correlation
Yan et al.^35^ (2017), China, English	Initial sample size: 40 conscious healthy volunteers, Randomised crossover study, Period of data collection not reported	Self-reported VAS	ANI (MetroDoloris)	Stimulus (cold pressor) after application of either vitamin E (VE) cream or lidocaine (LIDO), with a washout period of 2 weeks	Final sample size: 40 volunteers (r = − 0.27, P = 0.017), weak negative correlation between ANI and VAS scores; AUC: VAS > 30 mm = 0.603; VAS > 60 mm = 0.673 ANI distinguishes severe pain better than mild pain
Theerth et al.^36^ (2018), India, English	Initial sample size: 60 patients, 18–65 years Parallel-group, randomised active-active trial, May 2015- October 2016	Self-reported NRS in the immediate postoperative period	ANI (MetroDoloris) continuously monitored throughout the intra-operative period and in the immediate postoperative period	Elective surgery: supra-tentorial craniotomy for brain tumours General anesthesia: fentanil/sevoflurane	Final sample size: 57 patients Spearman correlation: r = 0.072, P = 0.617 No correlation was observed between the postoperative NRS Score and the postoperative ANIm values
Lee et al.^37^ (2019), Korea, English	Initial sample size: 201 patients, ASA I or II 20–79 years, Observational study, October 2014-October 2016	Self-reported NRS Recorded before surgery	ANI (MetroDoloris) recorded for10 min in the operating room before surgery and in PACU after surgery also for 10 min, SPI (Carescape B850; GE Healthcare, Milwaukee, WI, USA) recorded simultaneously as ANI	Elective surgery: thyroid, breast, or abdominal; General anesthesia: propofol /sevoflurane, remifentanil was infused intraoperatively	Final sample size: 192 patients Pearson correlation: (r = − 0.288, ANI = − 1.3 × NRS + 72.7, P < 0.001) weak relationships were observed between NRS and ANI values; AUC = 0.67, CI 0.62- 0.73 (P < 0.0001) Sensitivity: 50% Specificity: 82% ANI failed to distinguish between moderate (3 < NRS ≤ 7) and severe (7 < NRS ≤ 10) pain, P = 0.740
Charier et al.^38^ (2019), France, English	Initial sample size: not reported, 18–91 years Observational study, November 2014- March 2015	Self-reported VAS as soon as patients demonstrate wakefulness	ANI (MetroDoloris) 4 min until equilibrium of the signal, Pupillary Light Reflex (PLR) recorded simultaneously, Variation Coefficient of Pupillary Diameter (VCPD) recorded simultaneously	Orthopaedics, endoscopy, otorhinolaryngology, digestive surgery, neuro-spinal surgery, gynaecology, urology, and vascular surgery, General anesthesia	Final sample size: 345 patients Weak correlation were observed between VAS and ANI: Pearson correlation: (r = − 0.15; P = 0.006) Weak negative correlation between ANI and VAS scores AUC: 0.39, CI: 0.33–0.45,P = 0.001; ANI < 40 was predictive of a VAS ≥ 4: Sensitivity: 0.91, specificity of 0.14, PPV = 0.8 NPV = 0.27
Abdullayev et al.^39^ (2019), Turkey, English/Portuguese	Initial sample size: 120 patients, ASA I and II 18–65 years, Prospective observational study, January-March 2017	Self-reported NRS 15 min after arrival in PACU	ANI (MetroDoloris) 15 min after arrival in PACU (simultaneously)	Any surgical procedure under halogenated-based anaesthesia with fentanyl or remifentanil	Final sample size: 107 patients Pearson correlation: (r = − 0.312, p = 0.001) A significant negative relationship was observed between ANI and NRS
Soral et al.^15^(2020), Turkey, English	Initial sample size: not reported, ASA I and II 18–70 years, Prospective cohort study Oct 2015 to Jun 2016	Self-reported NRS	ANI (MetroDoloris) In Group A	Elective colonoscopy under sedo-analgesia ketamine, propofol and remifentanil Group A-remifentanil infusions, whereas in Group C- analgesic requirements were met according to the attending anaesthetist's intention	Final sample size: 102 patients Pearson correlation: (r = − 0.402, p = 0.003) Significant negative correlation between ANI and NRS scores of Group A patients at minute 0
Koprulu et al.^40^, Turkey, English	Initial sample size: 36 patients ASA I and II 18–75 years, May–August 2018	Self-reported NRS Recorded with 10 min of the admission of the patients to PACU	ANI (MetroDoloris) Recorded immediately before extubation in the operating room and after extubation in the PACU	Laparoscopic cholecystectomy; Sevoflurane/remifentanil anaesthesia	Final sample size: 36 patients Pearson correlation: Preextubation NRS/ANI correlation: Group I—NRS ≤ 3 (r = 0.016) Group II—NRS 4–6 (r = − 0.286) Group III—NRS ≥ 7 (r = − 0.293); Postextubation NRS/ANI correlation: Group I—NRS ≤ 3 (r = 0,135 ) Group II—NRS 4–6 (r = − 0.069) Group III—NRS ≥ 7 (r = − 0.290) Weak correlation between the NRS and ANI of all patient groups

*ANI* Analgesia Nociception Index, *ASA* American Society of Anesthesiologists, *AUC* area under the curve, *CI* confidence interval, *NPV* negative predictive value, *NRS* numerical rating scale, *PACU* post-anesthesia care unit, *PLR* pupillary light reflex, *PPV* positive predictive value, *ROC* receiver operating characteristics, *SD* standard deviation, *SE* standard error, *SEM* standard error of the mean, *SPI* surgical plethysmographic index, *VAS* visual analogue scale, *VCPD* variation coefficient of pupillary diameter.

### Risk of bias of individual studies

Two review authors (DAB, LRC) independently carried out the quality assessment using the University of Adelaide critical appraisal checklists for diagnostic test accuracy studies^27^ and analytical cross-sectional studies^28^. Any disagreement between the review authors over the risk of bias in individual studies was resolved by a third review author (LGA). For both tools, each item could be answered with yes (low risk of bias), no (high risk of bias), unclear (unclear risk of bias), or not applicable.

### Diagnostic accuracy measures

Accuracy and correlation measures were the outcomes. Sensitivity, specificity, positive predictive value, negative predictive value, area under the curve (AUC), and receiver operating characteristics (ROC) were used to measure diagnostic accuracy. Correlation between the ANI index and the self-reported pain diagnosis measures included r-values, *p* values, and confidence intervals (CI).

### Synthesis of results and subgroup analysis

Articles included that were methodologically homogeneous were incorporated into meta-analysis. Subgroup analyses were conducted considering the subjective pain measure used, medical procedures or electrical stimulus applied. Correlation analyses between ANI and subjective measures were conducted.

Statistical heterogeneity of the analyses was assessed using the I^2^ statistics. In the meta-analysis with an I^2^ higher than 40%, the random-effect model was used. In the meta-analysis with an I^2^ lower than 40%, the fixed-effect model was used^29^. Meta-analyses were conducted with the MedCalc statistical software version 19.2.6 (MedCalc Software bv, Ostend, Belgium; https://www.medcalc.org; 2020). Correlation coefficients (r values) and confidence intervals (CI) were provided.

## Results

### Study selection

**Figure 1:**
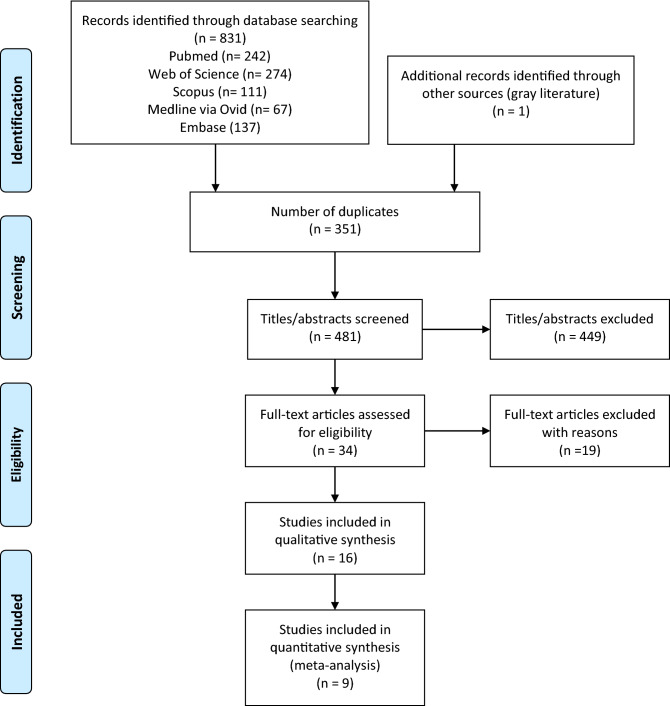
Flow chart depicting the search process.

Eight hundred thirty-two references were identified across the five electronic databases and the grey literature. Following the removal of 351 duplicate hits, 481 titles/abstracts were screened in Phase 1. The full texts of 34 references were retrieved, and the eligibility criteria were applied in Phase 2. Following the evaluation, 16 articles^15,16,22,30–42^, assessing 1.602 individuals, fulfilled the eligibility criteria and were included in this study (Fig. 1). The complete reference of the 19 articles in Phase 2 and the reasons for exclusion are presented (Supplementary Appendix A).

### Study characteristics

Among the 16 articles included, six reported accuracy and correlation measures^16,22,37,38,41,42^, seven only correlation tests^15,32,33,35,36,39,40^ and three reported accuracy measures exclusively^30,31,34^ (Table 1).

Three articles indicated the calculation of the sample size^36–38^. One was a pilot study conducted with French women during labor^22^, and another was carried out with individuals being treated for burn wounds^34^. Three articles were on conscious and healthy volunteers^32,33,35^, ten about aware patients after procedures under general anaesthesia^16,30,31,36–42^, and one in patients on sedo-analgesia (no premedication was administered before the procedure)^15^.

The self-reported subjective measures used to compare with ANI were the Visual Analogue Scale (VAS)^22,35,38,42^ and the Numerical Rating Scale (NRS)^15,16,30–34,36,37,39–41^. Three studies^34,37,38^ used objective measures other than ANI: cardiovascular depth of analgesia (CARDEAN)^34^, Surgical Plethysmographic Index (SPI)^37^, the pupillary light reflex (PLR)^37^, and the variation coefficient of pupillary diameter (VCPD)^38^.

### Risk of bias in individual studies

The methodological quality evaluation is summarised in Tables 2 and 3 (Supplementary Appendices B and C). The domain judged as having the highest risk of bias in accuracy studies was blinding the index test results concerning the reference standard because these results had not been cited or the test had not been performed.

Three correlation studies presented a high risk of bias in identifying confounding factors and strategies to deal with them^15,38,41^, while one study^34^ exhibited a high risk of bias in four items.

### Results of individual studies

ANI performed well to detect moderate to severe pain upon arrival in the Post-Anaesthesia Care Unit (PACU), which was improved with propofol-based (AUC = 0.93) in comparison with halogenated-based anaesthesiaUC = 0.82)^30^. Likewise, Boselli et al.^31^, demonstrated a high negative predictive value of ANI: ANI ≥ 50, predicting that 92% of patients had appropriate analgesia (NRS ≤ 3) upon arrival in PACU for orthopaedic surgery (AUC = 0.93, 95% CI 0.86–0.97) and otolaryngology surgery (AUC = 0.83, 95% CI 0.75–0.90). ANI measures correlated well with subjective NRS scores in the postoperative period after using volatile agents and opioid-based anaesthesia in another study^39^. In two other studies, the measure used in a similar scenario was VAS. Jeanne et al.^42^ evidenced no correlation between ANI and VAS scores (Spearman rank test, r^2^ = − 0.164, P=0.25) in total knee replacement orthopaedic surgery. Charier et al.^38^ also found a similar weak negative correlation (Pearson correlation, r = − 0.15; P=0.006) in surgeries whose general anaesthesia and postoperative analgesia protocols had been left to the anaesthetist's discretion.

ANI was strongly correlated with VAS in postpartum women (p < 0.0001), in particular before epidural analgesia^22^ and presented a weak negative correlation (r = − 0.15). Two studies reported that ANI did not reflect different states of moderate to severe pain measured after sevoflurane-based general anaesthesia in adults^16,37^, revealing low sensitivity and specificity to detect the difference between NRS 0 and NRS 6–10 (AUC = 0.43) in one study^16^. In patients who had undergone colonoscopy under sedo-analgesia, ANI correlated significantly with NRS (r = − 0.402)^15^.

One study did not obtain satisfactory results when correlating ANI with NRS in three different groups for pain intensity (group I: NRS ≤ 3, group II: NRS 4–6, group III: NRS ≥ 7) in patients who had undergone laparoscopic cholecystectomies under sevoflurane/remifentanil anaesthesia^40^, and no correlation was observed between the postoperative NRS score and the postoperative ANIm values in elective supratentorial tumour surgery^36^.

Papaioannou et al.^34^, demonstrated considerable sensitivity (67%) and specificity (70%) of ANI in predicting pain. Furthermore, the accuracy increased when associated with another measure (CARDEAN). Thus, ANI was fit to measure nociception in a group of conscious burnt patients under analgesic effects during wound care procedures.

One study showed no correlation between ANI minima and NRS in individuals submitted to unexpected electrical pain or expected electrical pain, indicating that ANI was neither a specific nor a robust measure to assess pain intensity compared with NRS in conscious men. There was no correlation between minima ANI and NRS when assessing painful stimuli (rs = − 0.01, P = 0.97)^33^. Issa et al.^32^, showed a weak correlation between ANI and NRS (Pearson, − 0.089; 95% CI − 0.192 to − 0.014; P = 0.045), suggesting that ANI was not specific for the assessment of pain intensity in alert volunteers. Yan et al.^36^, evaluated conscious, healthy volunteers with a cold pressor simulator, showing that the correlation between ANI and VAS was negative and weak (r = − 0.27 and P = 0.017).

### Synthesis of results and subgroup analysis

Nine studies were incorporated into a meta-analysis. Two subgroup analyses of correlation between ANI and NRS were feasible: (1) data of studies assessing conscious individuals who had undergone medical procedures under general anaesthesia were pooled; (2) data of studies evaluating participants submitted to electrical stimulus were pooled.

**Figure 2:**
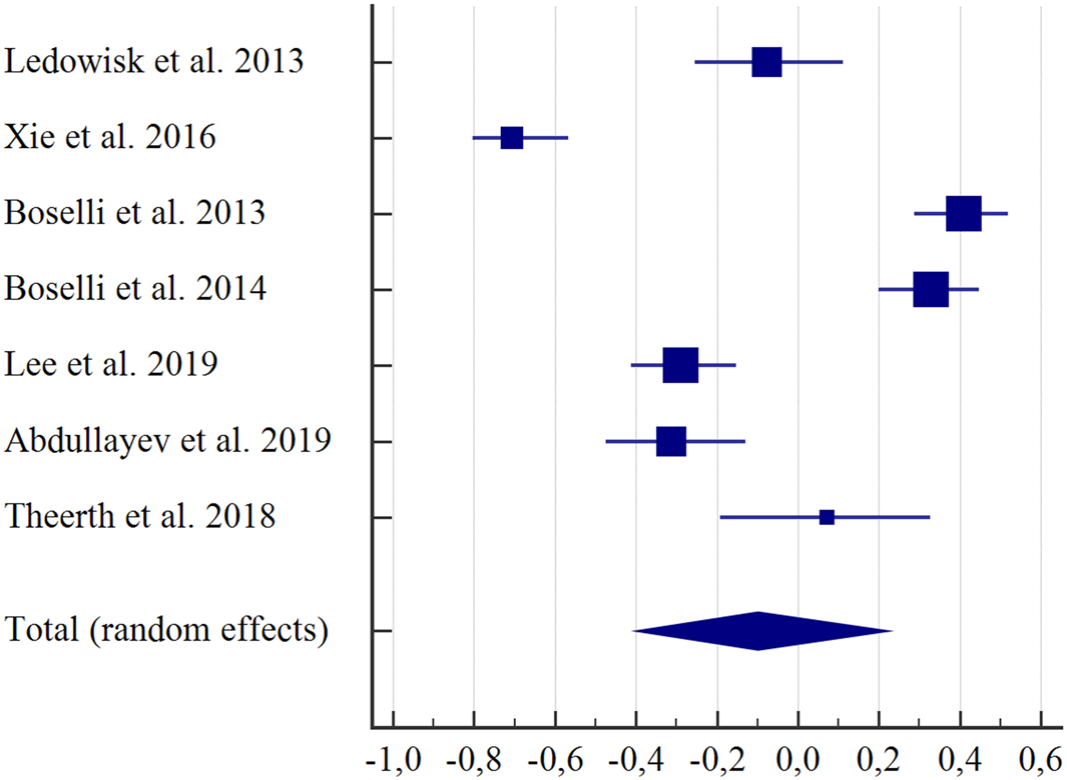
Subgroup correlation analysis between ANI and NRS in individuals submitted to medical procedures under general anaesthesia (r = − 0.0984, CI = − 0.397 to 0.220, I^2^ = 95.82%). The random-effect model was used. MedCalc Statistical Software version 19.2.6 (MedCalc Software bv, Ostend, Belgium; https://www.medcalc.org; 2020).

**Figure 3:**
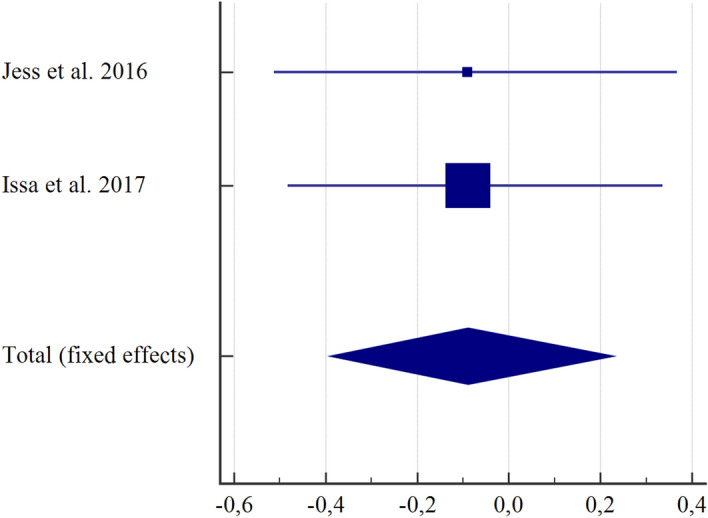
Subgroup correlation analysis between ANI and NRS in individuals submitted to electrical stimulus (r = − 0.089; CI = − 0.390 to 0.228, I^2^ = 0%). The fixed-effect model was used. MedCalc Statistical Software version 19.2.6 (MedCalc Software bv, Ostend, Belgium; https://www.medcalc.org; 2020).

In the first subgroup analysis of correlation, seven studies^16,30,31,36,37,39,41^ were incorporated. This subgroup demonstrated a weak negative correlation between ANI and NRS (r = − 0.0984, CI  − 0.397 to 0.220, I^2^ = 95.82%). The random-effect model was used (Fig. 2). The second subgroup analysis^32,33^ compared the ANI and the NRS in individuals who had been submitted to electrical stimulus and showed a weak negative correlation (r = − 0.089; CI = − 0.390 to 0.228, I^2^ = 0%). The fixed-effect model was used (Fig. 3).

## Discussion

This systematic review and meta-analysis demonstrated that nociception assessed through ANI had a weak and negative correlation with subjective self-reported measures of pain in conscious individuals, i.e., those undergoing medical procedures or submitted to painful experimental stimuli. However, good accuracy of the ANI as compared with NRS was observed in some studies^30,31,41^.

The current definition of pain by the International Association for the Study of Pain (IASP) (2020) is: "An unpleasant sensory and emotional experience associated with actual or potential tissue damage, or described in terms of such damage"^43^. For nociception, the concept is “The neural process of encoding noxious stimuli. Note: Consequences of encoding may be autonomic (e.g., elevated blood pressure) or behavioural (motor withdrawal reflex or more complex nocifensive behaviour). Pain sensation is not necessarily implied”^44^. These definitions strongly underline the influence of stress and emotions in modifying the correlation of nociception and pain assessment in awake individuals after surgical procedures or painful stimuli. The nature of pain is multifactorial^45^. Nociception depends on the trigger, and pain is clearly defined as a subjective experience^46^.

The satisfactory ANI's accuracy reported by Boselli et al.^30^ and Boselli et al.^31^ in the post-operative period should be interpreted with attention. Such findings may be clinically relevant because their results suggest that ANI can support practitioners to assess pain in the surgical setting and consequently allow a more reliable prescription of medications during and after surgical procedures. Thus, the use of ANI in the PACU may be potentially appropriate since inadequate management of pain in the postoperative period leads to undesirable results during the patient's recovery^47^.

On the other hand, the large variability in the results of correlation between ANI (objective measure) and NRS (subjective measure) in participants in the postoperative period of general anaesthesia proved by meta-analysis provides power to self-report measures of pain as the “gold standard” for deciding on analgesic complementation in conscious patients.

Our findings underline the influence of anaesthetic agents on ANI scores. However, there is no consensus on which anaesthetic agent would improve the correlation of ANI with subjective pain measures, possibly because the studies have compared different types of anaesthetics beside their relevant heterogeneity^16,30,31,36,37,39,41^. The anaesthetic agent and the drug consumption for pain control during the surgical procedure (remifentanil, fentanyl, sevoflurane, propofol, halogenated)^36–42,47–49^ or the technique (spinal, regional, or general anaesthesia)^22,50^ may influence the ANS regulation and alter the response of HRV to nociception.

Another vital point is whether the patients were conscious when answering about their pain. Factors such as the patient’s level of awareness and perception of the situation may also impact the final result of pain assessment^39^. Different surgical procedures^36,37,39,42^ and drugs' residual effect^36^ should also be taken into account in assessing pain. It is worth mentioning the negative correlation found in one included study, in which the patients exhibited spontaneous breathing during labour^22^.

As evidenced by studies included in the second subgroup analysis, the subjectivity of pain may impair the ANI assessment in individuals exposed to electrical stimuli^32,33^. The ANS is an essential regulator of heart rate. The transition in the time between two heartbeats is designated as HRV. It provides reliable information about the interaction of the sympathetic and parasympathetic nervous systems^51^. Parasympathetic activity is dominant in resting conditions, such as relaxation and sleep, whereas sympathetic activity increases heart rate and blood pressure in situations requiring energy expenditure. Their interaction is known as the sympathetic-vagal balance of the ANS. ANS balance and its conditions are reflected in HRV, which refers to short- and long-term heart rate variations due to several states, including emotional issues^52–56^. The sympathovagal balance is influenced by arousal, emotions, medications, and drugs used intraoperatively. Some of these factors, such as arousal and feelings, are more evident in conscious patients.

A weak correlation has made us reflect on the statistical analysis of the included studies whose authors had evaluated the agreement between two tests. Correlation analysis, the measure used by the majority, may be a powerless statistic. Therefore, in future research, accuracy measures or the Bland–Altman plot test should be used^57^.

According to a narrative review, measuring nociception in clinical settings is practically unfeasible. Still, it would be desirable for patients under general anaesthesia or unable to communicate to prevent acute postoperative pain. The authors conclude that no device has its usefulness justified in practice^5^. However, a recent systematised review described the validity of ANI for nociception assessment in anesthetised patients undergoing surgery and reported considerable changes in ANI values were found in response to nociceptive stimuli at different opioid concentrations and higher ANI values were noted during nociceptive stimuli^58^.

The studies included in our review used two Patient-Reported Outcome Measures (PROMS): VAS^22,35,38,42^ and NRS^16,30–34,36,37,39–41^. According to the FDA (Food and Drug Administration), a “PRO (Patient-Reported Outcome) is any report of the status of a patient's health condition that comes directly from the patient, without interpretation of the patient's response by a clinician or anyone else”. Therefore, the accuracy of ANI was assessed according to PROs^59^.

The present study has limitations. Although individuals in the included studies were conscious and reported their pain, the pain stimuli assessed were quite different. Women in labour, patients treated for burns, various elective surgeries, and patients who had received electrical stimuli may exhibit different responses to pain, taking into account the subjective pattern of pain and the influence of nociceptive stimuli. This study provided two subgroups of meta-analysis, but it is necessary to consider that, individually, some included studies^30,31,41^ demonstrated adequate accuracy and correlation of ANI with subjective measures of pain.

The strengths of this systematic review and meta-analysis include a comprehensive literature search through major electronic databases and grey literature, adherence to the PRISMA guidelines, and the inclusion of the highest number of studies published on this topic. Finally, data extraction, evaluation of outcomes, and the risk of bias assessment were all performed in duplicate. One limitation is the methodological heterogeneity among the included studies with different designs, precluding additional aggregated analyses. Among the included studies, only nine showed homogeneity. Due to differences regarding the setting where the studies had been conducted, a unique meta-analysis was unfeasible. Analyses were conducted in two subgroups; in one subgroup, data of only two studies were aggregated. According to the literature, quantitative analyses (even those with a few studies) represent a powerful tool to summarise data and increase sample size, allowing the researchers to obtain more reliable estimates. Nevertheless, the findings of those quantitative analyses should be interpreted with caution due to shortcomings of data that have been aggregated, such as studies' risk of bias, publication bias, and small-study effect^60,61^.

## Conclusion

There was a weak correlation between the subjective pain scales and the Analgesia and Nociception Index, i.e., a part of pain self-report is explained by nociception assessed through ANI. Therefore, in the perioperative period, fully or partially conscious children or other individuals, who cannot self-report their pain, might benefit from using ANI during health procedures.

## Supplementary Information

Supplementary Information 1.

Supplementary Information 2.

Supplementary Information 3.

## Data Availability

All data generated or analysed during this study are included in this published article (and its Supplementary Information files).
