# Supplementary material for: Comparison between Analgesia Nociception Index (ANI) and self-reported measures for diagnosing pain in conscious individuals: a systematic review and meta-analysis
Source: Sci Rep. 2022 Feb 21;12:2862. doi: 10.1038/s41598-022-06993-z (PMC8860998; doi:10.1038/s41598-022-06993-z)
Supplement: Supplementary file 1 — Supplementary Information 1. [file 41598_2022_6993_MOESM1_ESM.docx]

Supplementary Appendix A

Supporting information: Articles excluded following full-text reading

1- Boselli, E. Interest of Analgesia/Nociception Index (ANI) for monitoring the relative parasympathetic tone in anesthetized and awake patients. *Douleurs*. **19**, 205-10. <https://doi.org/10.1016/j.douler.2018.07.008> (2018).

Reason for exclusion: This study is a literature review.

2- Boselli, E., Musellec, H., Bernard, F. *et al*. Effects of conversational hypnosis on relative parasympathetic tone and patient comfort during axillary brachial plexus blocks for ambulatory upper limb surgery: A Quasiexperimental Pilot Study. [*Int J Clin Exp Hypn*](https://www.ncbi.nlm.nih.gov/pubmed/?term=EFFECTS+OF+CONVERSATIONAL+HYPNOSIS+ON+RELATIVE+PARASYMPATHETIC+TONE+AND+PATIENT+COMFORT+DURING+AXILLARY+BRACHIAL+PLEXUS+BLOCKS+FOR+AMBULATORY+UPPER+LIMB+SURGERY%3AA+Quasiexperimental+Pilot+Study). **66**: 34-46. DOI: 10.1080/00207144.2018.1421355 (2018)

Reason for exclusion: This study does not refers to pain. Patient’s comfort was evaluated.

3- Boselli, E., Bouvet, L. & Allaouchiche, B. Analgesia monitoring using Analgesia/Nociception Index: Results of clinical studies in awake and anesthetized patients. *Prat Anesth Reanim*. **19**: 78-86. <https://doi.org/10.1016/j.pratan.2015.03.006> (2015)

Reason for exclusion: This study is a literature review.

4- Boselli, E., Begou, G., Bouvet, L. *et al*. Prediction of immediate postoperative pain with Analgesia/Nociception Index (ANI): observational study. *Ann Fr Anesth Reanim*. **32**: 351-2 (2013).

Reason for exclusion: This study is a conference abstract.

5- Boselli, E. & Jeanne, M. Analgesia/nociception index for the assessment of acute postoperative pain. *Br J Anaesth*. **112**: 936-37 (2014).

Reason for exclusion: This study is an author's comment.

6- Chanques, G., Tarri, T., Ride, A. *et al*. Analgesia nociception index for the assessment of pain in critically ill patients: a diagnostic accuracy study. *Br J Anaesth.* **119:** 812-20. DOI: [10.1093/bja/aex210](https://doi.org/10.1093/bja/aex210) (2017)

Reason for exclusion: This study is a study conducted with unconscious patients. This study also does not correlate ANI with a self-report scale or questionnaire.

7- Hochhausen, N., Ritter, M., Kony, M. *et al*. Support of acute pain therapy by Analgesia Nociception Index (ANI) in Postanesthesia Care Unit (PACU). *Acta Anaesthesiol Scand*. **59** (2015).

Reason for exclusion: This study is a conference abstract.

8- Ledowski, T. Analgesia-nociception index. *Br J Anaesth*. **112**: 937 (2014).

Reason for exclusion: This is an author’s comment.

9- Ledowski, T. Monitoring nociception - Getting 'there yet' might be easier with a road map. *Br J Anaesth*. **119:** 716-7 (2017).

Reason for exclusion: This study is an author’s comment.

10- Martin, C., Robert, T., Montravers, P. *et al*. Evaluation of preoperative anxiety by Analgesia Nociception Index. *Ann Fr Anesth Reanim*. **32**: 347-52 (2013).

Reason for exclusion: This study used a questionnaire that evaluates stress rather than pain.

11- Six, S., Laureys, S., Poelaert, J. *et al*. Comfort in palliative sedation (Compas): a transdisciplinary mixed method study protocol for linking objective assessments to subjective experiences. *BMC Palliat Care*. **17**,62-8. DOI: 10.1186/s12904-018-0316-2 (2018).

Reason for exclusion: In this study, the patients were sedated and did not answer any self report scale or questionnaire.

12- Szental, J.A., Webb, A., Weeraratne, C. *et al*. Postoperative pain after laparoscopic cholecystectomy is not reduced by intraoperative analgesia guided by analgesia nociception index (ANIÂ®) monitoring: A randomized clinical trial. *Br J Anaesth*. **114**: 640-5. <https://doi.org/10.1093/bja/aeu411> (2015).

Reason for exclusion: In this study, the author did not compare ANI with any scale or questionnaire for pain evaluation.

13- Eisenried, A., Akhbardeh, A., Yeomans, D.C. *et al*. Objective measurement of pain perception in volunteers and anesthetized patients. *Anesth Analg*. **122**: 5 (S352) (2016).

Reason for exclusion: This study was an abstract of Posters presented at the 2016 Annual Meeting of the International Anesthesia Research Society.

14- Bouzit, Z., Grati, A.H. & Dhonneur, G.F. Does apnea affects the reliability of Analgesia Nociception Index (ANI), as a monitor of nociception? *Annals of Intensive Care*. **7**: 75 (2017).

Reason for exclusion: This study does not correlate ANI with VAS nor shows ANI’s accuracy.

15- Ledowski, T., Tiong, W.S., Lee, C., Wong, B., Fiori, T. & Parker, N. Is acute postoperative pain reflected by a change in parasympathetic cardiac tone? Evaluation of the Analgesia Nociception Index. *Anaesth Intens Care*. **40**:1064 (2012).

Reason for exclusion: This study is an Australian Society of Anaesthetists National Scientific Congress abstract paper.

16- Gazi, M., Abitağaoğlu, S., Turan, G., Köksal, C., Akgün, F.N. & Arı, D.E. Evaluation of the effects of dexmedetomidine and remifentanil on pain with the analgesia nociception index in the perioperative period in hysteroscopies under general anesthesia: A randomized prospective study. [*Saudi Med J*](https://www.ncbi.nlm.nih.gov/pubmed/30284585). **39**: 1017-22. DOI: [10.15537/smj.2018.10.23098](https://dx.doi.org/10.15537%2Fsmj.2018.10.23098) (2018).

Reason for exclusion: This study does not correlate ANI with VAS nor shows ANI’s accuracy.

17- Bernard, T., Gaillard, T., Gergaud, S. & Lasocki, S. Analgesia Nociception Index-based analgesia protocol reduces pain during nursing in critically ill patients: a before/after study. *Intensive Care Medicine Experimental*. **5**:132 (2017).

Reason for exclusion: This study is a meeting abstract.

18- Choi, B.M., Shin, H., Lee, J.H., Bang, J.Y., Lee, E.K. & Noh, G.J. Performance of the Surgical Pleth Index and Analgesia Nociception Index in Healthy Volunteers and Parturients. *Frontiers in physiology*. **12**: 218 (2021).

Reason for exclusion: This study not correlate ANI with NRS nor shows ANI’s accuracy.

19- Puig, M.A., Alonso-Prieto M., Miró, J., Torres-Luna, R., de Sabando, D.P.L. & Reinoso-Barbero F. The Association Between Pain Relief Using Video Games and an Increase in Vagal Tone in Children With Cancer: Analytic Observational Study With a Quasi-Experimental Pre/Posttest Methodology. *Journal of medical Internet research.* **22**(3), e16013 (2020).

Reason for exclusion: This study not correlate ANI with NRS nor shows ANI’s accuracy.
