# Supplementary material for: Comparison between Analgesia Nociception Index (ANI) and self-reported measures for diagnosing pain in conscious individuals: a systematic review and meta-analysis
Source: Sci Rep. 2022 Feb 21;12:2862. doi: 10.1038/s41598-022-06993-z (PMC8860998; doi:10.1038/s41598-022-06993-z)
Supplement: Supplementary file 2 — Supplementary Information 2. [file 41598_2022_6993_MOESM2_ESM.docx]

Supplementary Appendix B

**Table 2**. Quality assessment of included studies using the University of Adelaide critical appraisal checklist for diagnostic test accuracy studies

|  | Patient selection | | | Index test | | | | | | |
| --- | --- | --- | --- | --- | --- | --- | --- | --- | --- | --- |
| Author, year | Was a consecutive or random sample of patients enrolled? | Was a case control design avoided? | Did the study avoid inappropriate exclusions? | Were the index test results interpreted without knowledge of the results of the reference standard? | If a threshold was used, was it pre-specified? | Is the reference standard likely to correctly classify the target condition? | Were the reference standard results interpreted without knowledge of the results of the index test? | Was there an appropriate interval between index test and reference standard? | Did all patients receive the same reference standard? | Were all patients included in the analysis? |
| Le Guen et al. (2012) | Low risk of bias | Low risk of bias | Low risk of bias | Low risk of bias | Low risk of bias | Low risk of bias | Unclear risk of bias * | Low risk of bias | Low risk of bias | Low risk of bias |
| Boselli et al. (2013) | Low risk of bias | Low risk of bias | Low risk of bias | Low risk of bias | Low risk of bias | Low risk of bias | Unclear risk of bias * | Low risk of bias | Low risk of bias | Low risk of bias |
| Ledowisk et al. (2013) | Low risk of bias | Low risk of bias | Low risk of bias | Unclear risk of bias* | Low risk of bias | Low risk of bias | Low risk of bias | Low risk of bias | Low risk of bias | Low risk of bias |
| Boselli et al. (2014) | Low risk of bias | Low risk of bias | Low risk of bias |  | Low risk of bias | Low risk of bias | Unclear risk of bias * | Low risk of bias | Low risk of bias | Low risk of bias |
| Jeanne et al. (2014) | Low risk of bias | Low risk of bias | Low risk of bias | Low risk of bias | Low risk of bias | Low risk of bias | Unclear risk of bias * | Low risk of bias | Low risk of bias | Low risk of bias |
| Papaioannou et al. (2016) | Low risk of bias | Low risk of bias | Low risk of bias | Unclear risk of bias* | High risk of bias | Low risk of bias | Unclear risk of bias * | Low risk of bias | Low risk of bias | Low risk of bias |
| Xie et al. (2016) | Unclear risk of bias* | Low risk of bias | Low risk of bias | Unclear risk of bias* | Unclear risk of bias* | Low risk of bias | Unclear risk of bias * | Low risk of bias | Low risk of bias | Low risk of bias |
| Yan et al. (2017) | Low risk of bias | Low risk of bias | Unclear riskof bias* | Unclear risk of bias* | High risk of bias | Low risk of bias | Unclear risk of bias * | Low risk of bias | Low risk of bias | Unclear risk of bias * |
| Lee et al. (2019) | Low risk of bias | Low risk of bias | Low risk of bias | Unclear risk of bias* | Low risk of bias | Low risk of bias | Unclear risk of bias* | Low risk of bias | Low risk of bias | Low risk of bias |
| Charier et al. (2019) | Low risk of bias | Low risk of bias | Low risk of bias | Low risk of bias | Low risk of bias | Low risk of bias | Low risk of bias | Low risk of bias | Low risk of bias | Low risk of bias |

* not specified/ not cited
