# Supplementary material for: Comparison between Analgesia Nociception Index (ANI) and self-reported measures for diagnosing pain in conscious individuals: a systematic review and meta-analysis
Source: Sci Rep. 2022 Feb 21;12:2862. doi: 10.1038/s41598-022-06993-z (PMC8860998; doi:10.1038/s41598-022-06993-z)
Supplement: Supplementary file 3 — Supplementary Information 3. [file 41598_2022_6993_MOESM3_ESM.docx]

Supplementary Appendix C

**Table 3**. Quality assessment of included studies using the University of Adelaide critical appraisal checklist for analytical cross-sectional studies

| Author, year | Were the criteria for inclusion in the sample clearly defined? | Were the study subjects and the setting described in detail? | Was the exposure measured in a valid and reliable way? | | Were objective, standard criteria used for measurement of the condition? | | Were confounding factors identified? | | Were strategies to deal with confounding factors stated? | | Were the outcomes measured in a valid and reliable way? | | Was appropriate statistical analysis used? | |  |  |  |
| --- | --- | --- | --- | --- | --- | --- | --- | --- | --- | --- | --- | --- | --- | --- | --- | --- | --- |
| Le Guen et al. (2012) | Low risk of bias | Low risk of bias | Low risk of bias | | Low risk of bias | | Low risk of bias | | Low risk of bias | | Low risk of bias | | Low risk of bias | |  |  |  |
| Boselli et al. (2013) | Low risk of bias | Low risk of bias | Low risk of bias | | Low risk of bias | | Low risk of bias | | Low risk of bias | | Low risk of bias | | Low risk of bias | |  |  |  |
| Ledowski et al. (2013) | Low risk of bias | Low risk of bias | | Low risk of bias | | Low risk of bias | | Low risk of bias | | Low risk of bias | | Low risk of bias | | Low risk of bias |  |  |  |
| Boselli et al. (2014) | Low risk of bias | Low risk of bias | | Low risk of bias | | Low risk of bias | | Low risk of bias | | Low risk of bias | | Low risk of bias | | Low risk of bias |  |  |  |
| Jeanne et al. (2014) | Low risk of bias | Low risk of bias | | Low risk of bias | | Low risk of bias | | Low risk of bias | | Unclear risk of bias* | | Low risk of bias | | Low risk of bias |  | |  |
| Jess et al. (2016) | Low risk of bias | Low risk of bias | | Low risk of bias | | Low risk of bias | | Low risk of bias | | Low risk of bias | | Low risk of bias | | Low risk of bias |  |  |  |
| Xie et al. (2016) | Unclear risk of bias* | Low risk of bias | | Low risk of bias | | Low risk of bias | | High risk of bias | | High risk of bias | | Low risk of bias | | Low risk of bias |  |  |  |
| Theerth et al. (2018) | Low risk of bias | Low risk of bias | | Low risk of bias | | Low risk of bias | | Low risk of bias | | Low risk of bias | | Low risk of bias | | Low risk of bias |  |  |  |
| Issa et al. (2017) | Low risk of bias | Low risk of bias | | Low risk of bias | | Low risk of bias | | Low risk of bias | | Low risk of bias | | Low risk of bias | | Low risk of bias |  |  |  |
| Yan et al. (2017) | High risk of bias | High risk of bias | | Low risk of bias | | Low risk of bias | | High risk of bias | | High risk of bias | | Low risk of bias | | Low risk of bias |  |  |  |
| Lee et al. (2019) | Low risk of bias | Low risk of bias | | Low risk of bias | | Low risk of bias | | Low risk of bias | | Low risk of bias | | Low risk of bias | | Low risk of bias |  |  |  |
| Charier et al. (2019) | Low risk of bias | Low risk of bias | | Low risk of bias | | Low risk of bias | | High risk of bias | | High risk of bias | | Unclear risk of bias* | | Low risk of bias |  |  |  |
| Abdullayev et al. (2019) | Low risk of bias | Low risk of bias | | Low risk of bias | | Low risk of bias | | Low risk of bias | | Low risk of bias | | Low risk of bias | | Low risk of bias |  |  |  |
| Koprulu et al. (2020) | Low risk of bias | Low risk of bias | | Low risk of bias | | Low risk of bias | | Low risk of bias | | Low risk of bias | | Low risk of bias | | Low risk of bias |  |  |  |
| Soral et al. (2020) | Low risk of bias | Low risk of bias | | Low risk of bias | | Low risk of bias | | High risk of bias | | High risk of bias | | Low risk of bias | | Low risk of bias |  |  |  |

* not specified/ not cited
